# One-line hybrid rice with high-efficiency synthetic apomixis and near-normal fertility

**DOI:** 10.1007/s00299-024-03154-6

**Published:** 2024-02-24

**Authors:** Junhao Dan, Yumei Xia, Yao Wang, Yijie Zhan, Junyou Tian, Ning Tang, Huafeng Deng, Mengliang Cao

**Affiliations:** 1 Long Ping Branch, College of Biology, Hunan University, Changsha, 410082 People’s Republic of China; 2 State Key Laboratory of Hybrid Rice, Hunan Hybrid Rice Research Center, Changsha, 410125 People’s Republic of China; 3 School of Breeding and Multiplication (Sanya Institute of Breeding and Multiplication), Hainan University, Sanya, 572025 People’s Republic of China; 4 National Center of Technology Innovation for Saline-Alkali Tolerant Rice in Sanya, Sanya, 572000 People’s Republic of China

**Keywords:** One-line hybrid rice, Apomixis, Multiple-embryos, Atavism

## Abstract

**Key message:**

High-frequency clonal seeds and near-normal fertility were obtained by engineering synthetic apomixis in hybrid rice.

**Abstract:**

The one-line strategy, with the advantage of unnecessary seed production, is the final stage for the hybrid rice development and can be achieved through the fixation of heterosis via artificially inducing apomixis. Recently, synthetic apomixis has been generated in rice by combining *MiMe* (Mitosis instead of Meiosis) with either the ectopic expression of *BABY BOOM* (*BBM1* or *BBM4*) or mutation of *MATRILINEAL* (*MTL*), resulting in over 95.00% of clonal seeds. However, the frequency of clonal seeds was only 29.20% when *AtDD45* promoter was used to drive *BBM1*. In addition, achieving both a high frequency of clonal seeds and near-normal fertility simultaneously had been elusive in earlier strategies. In this study, using *AtDD45* promoter to drive *BBM1* expression in combination with the *MiMe* mutant resulted in the apomixis frequency as high as 98.70%. Even more, employing fusion promoters (*AtMYB98*_*AtDD1*_*OsECA1-like1*) to drive *WUS* expression in combination with *pAtDD45*:*BBM1* and *MiMe* could produce clonal seeds at rates of up to 98.21%, the highest seed setting rate reached to 83.67%. Multiple-embryos were observed in clonal lines at a frequency ranging from 3.37% to 60.99%. Transmission of the high frequency of apomixis through skipped generations (atavism) was identified in two clonal lines, even though it remained stable in the majority of clonal lines. These findings significantly advance the pursuit of fixed heterosis in rice through synthetic apomixis, edging closer to its agricultural application.

**Supplementary Information:**

The online version contains supplementary material available at 10.1007/s00299-024-03154-6.

## Introduction

Hybrid rice (*Oryza sativa L.*), with significant vigor in traits including yield, adaptability, and biotic and abiotic stress tolerance, have broken the yield ceiling of their inbred counterpart and have been extended exclusively in China and abroad (Birchler et al. 2010). Since Professor Longping Yuan’s team discovered *O. rufipogon*, a wild male sterile plant, in Hainan Province, China, in 1970, and developed successfully hybrid rice in 1973 (Yuan 1977), China has managed to feed nearly 20% of the world’s population with less than 9%of the world’s arable land with benefit from this technology. Outside of China, hybrid rice has also been introduced to nearly 70 countries across five continents, e.g., America, Vietnam, Pakistan, India, Philippines. However, hybrid seed production is costly and laborious (Grossniklaus et al. 2004), because F_1_ hybrid seeds must be renewed at every crop season. In 1987, Longping Yuan proposed three phases of the development of hybrid rice breeding, each using a particular methodology: the three-line method or Cytoplasmic Male Sterility (CMS) system; the two-line method or Thermosensitive or Photoperiod-sensitive Genic Male Sterile (TGMS or PGMS) system; the one-line method or apomixis system (Yuan 1987). The former two approaches have been most successfully applied in hybrid breeding (Mou 2016), but the one-line method is still under investigation.

Until recent years, an alternative to male sterility systems has emerged by engineering synthetic apomixis in plants which makes one-line hybrid rice possible. Sundaresan’s team ectopically expressed the *BBM1* gene in rice egg cells in combination with *MiMe* and obtained approximately 29.20% of clonal seeds (Khanday et al. 2019). Guiderdoni’s team produced more than 95.00% of clonal seeds using *MiMe* mutants and *AtECS* promoter-driven *BBM1* in a single step (Vernet et al. 2022). Kejian Wang’s team obtained 5.23% of clonal seeds by simultaneously editing *REC8*, *PAIR1*, *OSD1*, and *MTL* genes in hybrid rice (Wang et al. 2019), and three years later, they used the same strategy to discover the descendant clonal plants remained stable (Liu et al. 2023). In 2023, Kejian Wang’s team obtained a high seed-setting rate (range 80.90–86.10%) of clonal diploid plants by combining *MiMe* with ectopic expression of *BBM4* in egg cells (Wei et al. 2023). Although the above studies represented important breakthroughs, the high clonal seeds-inducing rate and the high seed-setting rate have not yet been achieved simultaneously.

In this study, we introduced all-in-one T-DNA constructs into commercial hybrid rice, combined *MiMe* with ectopic expression of *BBM1*, which was driven by *AtDD45* promoter or fusion promoter, a high frequency of apomixis, multiple-embryos, and near-normal fertility were obtained. Atavism (transmission through skipped generations) of the high frequency of apomixis was identified in two clonal lines, while the majority of clonal lines remained stable.

## Materials and methods

### Plant materials and growth conditions

Commercial cultivars Yongyou4949 (YY4) (Japonica/indica hybrid rice released in 2016 in China) were used in this study. Transgenic plants (T_1_, T_2_, T_3_, T_4_, and T_5_ generations), YY4 hybrid, and F_2_ progenies were grown as usual.

The seeds were randomly sampled and put into seed bags, placed in a covered foam box filled with sterile water, and soaked at 37 °C for 24 h. Then, the seeds were wrapped with a moist towel and cultivated for 2–3 days. The seeds were then placed in a petri dish and covered with three layers of sterilized filter paper. Afterward, an appropriate amount of sterile water was added, and germination was monitored after the seeds were cultivated in light at 28 °C for 6 days.

### Plasmid construction and rice transformation

A CRISPR/Cas9 knockout expression vector p24MiMe (sg*MiMe*), designed to generate *MiMe* mutants by editing the *OsOSD1* (Os02g37850), *PAIR1* (Os03g01590), and *OsREC8* (Os05g50410) genes (the target sites were listed in Table S5), was constructed using a method described previously (Ma et al. 2015). The expression cassette, including the coding sequence (CDS) of embryogenic gene *BBM1* driven by the egg-specific promoter *AtDD45* and terminated by *Nos* terminator (Steffen et al. 2007), was synthesized and cloned into the p24MiMe vector, the produced sg*MiMe*_*pAtDD45:BBM1* was renamed p63C vector (GenScript Biotech Corp Co., Ltd., Nanjing). The p94C (‘sg*MiMe*’_*pAtDD45:BBM1*) vector with modified gRNAs of three *MiMe* genes was constructed to increase editing efficiency (Edgene Biot Co., Ltd., Wuhan, the target sites were listed in Table S5). The expression cassette, containing the fusion promoters (*Arabidopsis MYB98* synergid cell-specific promoter; *Arabidopsis DD1* antipodal cell-specific promoter; rice *ECA1*-*like1* egg cell-specific promoter) (Susaki et al. 2021) driving the *AtWUS* CDS and terminated by *PINII* terminator, was synthesized and cloned into the p94C vector, the produced ‘sg*MiMe*’_*pAtMYB98*^*+*^* pAtDD1*^*+*^* pOsECA1*-*like1:WUS*_*pAtDD45:BBM1* was renamed p95C vector (GenScript Biotech Corp Co., Ltd., Nanjing). These vectors were subsequently introduced into *EHA105* competent cells and transformed into YY4 hybrid, afterwards, calli induction and plant regeneration were performed according to methods described previously (Xia et al. 2019).

### Identification of mutants

Genomic DNA from the leaf of transgenic and control plants was extracted using the cetyltrimethylammonium bromide (CTAB) method (Doyle and Doyle 1990). PCR amplification of the *OsOSD1*, *PAIR1*, and *OsREC8* genes was performed using 2 × Taq PCR Mix (primers were listed in Table S6). The amplified fragments were sequenced by the Sanger method, and the sequencing results were compared and analyzed by Sequencher 5.4.6 software (Tsingke Biotechnology Co., Ltd., Beijing).

### Genotyping by pinpoint sequencing of multiplex PCR products

Batch Primer 3 software was used to design specific primers for target regions. These specific primers were used to amplify target interval sequences via multiple PCR, and the amplified target interval sequences were captured and enriched to construct a sequencing library. The samples were then sequenced via high-throughput sequencing. The raw data were filtered with FastQC 0.11.4 software to obtain clean reads, and the Burrows-Wheeler Alignment tool (BWA) was used to compare the clean sequences with the rice reference genome sequence. The single-nucleotide polymorphisms (SNPs) among the clean reads were detected and filtered by FreeBayes software (Higentec Co., Ltd., Changsha).

### Flow cytometry determination

The ploidy of transgenic and control plants was determined (Ploidy Expert, Beijing) by estimating nuclear DNA content using flow cytometry (NovoCyte 3005), following the guidelines of the reagent kit (CyStain^TM^PI Absolute P).

### Agronomic traits determinations and data analysis

Transgenic plants were grown at the Sanya transgenic base in Hainan and Changsha transgenic base in Hunan, China. The following agronomic traits were investigated at mature stage: plant height, number of panicles, panicle length, seed setting rate (filled grains/total grains), one-thousand grain weight, grain length, and grain width. For these agronomic traits, we used Duncan’s test with a significance of 0.05%. The SPASS software was used to analyze data.

### Cytological observations of rice embryo sacs

Wild-type and transgenic florets at different stages were collected and fixed in FAA solution, kept at room temperature for 24 h, washed with 50% ethanol, and then stored at 4 °C in 70% ethanol. Embryo sacs were prepared according to a previous method (Huang et al. 2017) and were scanned by a confocal laser scanning microscope (Zeiss LSM880) with excitation wavelength 543 nm and the emission wavelength 550–630 nm.

## Results

### High efficiency of apomixis and near-normal fertility resulted from the ectopic expression of *BBM1* driven by *AtDD45 *or fusion promoter combined with *MiMe* in hybrid rice

**Fig. 1:**
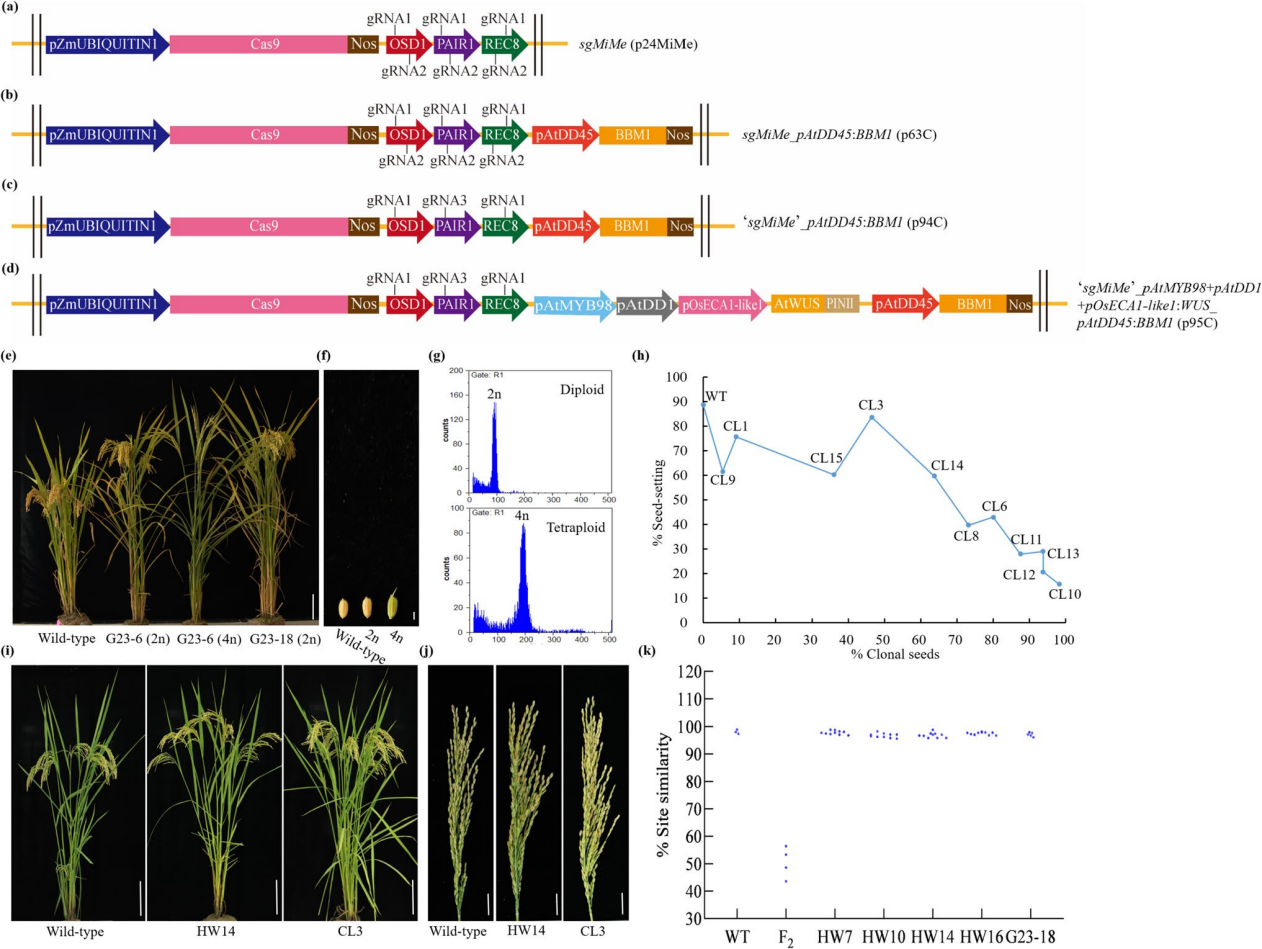
Ploidy, genotype, and phenotype of progeny plants of transformations in hybrid rice. **a** Schematic diagram of the T-DNA structure sg*MiMe* (p24MiMe) targeting *OsOSD1*, *PAIR1,* and *OsREC8* (*MiMe*). pZmUBIQUITIN1, *Zea mays UBIQUITIN 1* promoter; Cas9, *Cas9* gene; Nos, nopaline synthase terminator. **b** Schematic diagram of the T-DNA construct sg*MiMe*_*pAtDD45*:*BBM1* (p63C) containing the *MiMe* and *pAtDD45:BBM1* gene expression cassettes. pAtDD45, egg cell-specific from *Arabidopsis*; BBM1, *BBM1* gene. **c** Schematic diagram of the T-DNA construct ‘sg*MiMe*’_*pAtDD45*:*BBM1* (p94C). Compared to p63C, p94C was created by altering the gRNAs of three *MiMe* genes to increase editing efficiency. **d** Schematic diagram of the T-DNA construct ‘sg*MiMe*’_*pAtMYB98_pAtDD1_pOsECA1-like1:WUS*_*pAtDD45*:*BBM1* (p95C). pAtMYB98, synergid cell-specific from *Arabidopsis*; pAtDD1, antipodal cell-specific from *Arabidopsis*; pOsECA1-like1, egg cell-specific from rice; WUS, *WUS* gene from *Arabidopsis*. PinII, the terminator of proteinase inhibitor II. **e** Plant morphology of line G23-6 and line G23-18 (p63C) were compared to wild-type. The sexual tetraploid plant displayed high sterility. Scale bars, 20 cm. **f** Comparison of the T_1_ seeds between line G23-6 (p63C) and wild-type. The seed of tetraploid showed a large size with awns. Scale bars, 2 mm. **g** Representative ploidy analysis of a tetraploid and diploid according to flow cytometry. **h** The frequency of clonal seeds and seed-setting of p95C events. In the T_1_ clonal lines of p95C, a high seed-setting rate was observed, but the clonal seed rate was low. Conversely, there was a high frequency of clonal seeds with a low seed-setting rate. Details were provided in Table 2. **i** Plants morphology of line HW14 (p94C) and line CL3 (p95C) were compared to wild-type. Scale bars, 20 cm. **j** Comparison of the panicles between T_1_ diploid clonal plants (p94C and p95C) and wild-type. Scale bars, 5 cm. **k** Genotyping by pinpoint sequencing of wild-type, F_2_ sexual progenies, T_1_ clonal lines of p94C and p63C. Compared to wild-type, the site similarity of F_2_ progeny plants ranged from 43.62% to 56.44% resulting from meiotic crossovers, while diploid clonal plants maintained a site similarity of more than 95.00% due to the absence of meiotic division

To generate clonal seeds through apomixis, four T-DNA constructs were transformed into the commercial hybrid rice YY4 using an *Agrobacterium*-mediated method. The first construct sg*MiMe* (p24MiMe) was designed to knock out *OsOSD1*, *PAIR1*, and *OsREC8* genes via CRISPR/Cas9, leading to mitosis instead of meiosis (*MiMe*) and production of diploid clonal gametes (Khanday et al. 2019; Mieulet et al. 2016; Wang et al. 2019) (Fig. 1a). The second construct sg*MiMe*_*pAtDD45*:*BBM1* (p63C) contained the *BBM1* gene driven by the *Arabidopsis* egg-cell-specific promoter *AtDD45* (*pAtDD45*:*BBM1*) created in p24MiMe background, inducing the autonomous embryonic development in *MiMe* mutant (Fig. 1b). The third construct ‘sg*MiMe*’_*pAtDD45*:*BBM1* (p94C) was created by altering the gRNAs of three *MiMe* genes in the p63C background to increase editing efficiency (Fig. 1c). The fourth construct ‘sg*MiMe*’_*pAtMYB98_pAtDD1_pOsECA1*-*like1:WUS*_*pAtDD45:BBM1* (p95C), created in p94C background, in addition carried *WUS* driven by the fusion promoter (*AtMYB98*_*AtDD1*_*OsECA1*-*like1*) (Susaki et al. 2021), a cassette triggering apomixis from synergid cells and antipodal cells beside of egg cell to enhance the efficiency of apomixis (Fig. 1d). In the T_0_ transgenic populations, 3 (*n* = 63, 4.76%), 10 (*n* = 235, 4.26%), 29 (*n* = 42, 69.05%), and 25 (*n* = 42, 59.52%) transformants were identified as triple mutants in the p24MiMe, p63C, p94C, and p95C, respectively. This suggests that modifying the gRNA of the three *MiMe* genes can significantly improve editing efficiency in hybrid rice.

For examining ploidy and seed-setting rate, a crucial agricultural trait for apomixis, 3, 2, 5, and 11 *MiMe* mutants were selected from the fertile T_0_ transformants of the p24MiMe, p63C, p94C, and p95C, respectively. Clonal diploid plants are easily distinguishable by phenotypic observation from tetraploid plants, characterized by high sterility and large, awned grains (Fig. 1e–f), which could be confirmed by flow cytometry analysis (Fig. 1g). First, a conflicted relationship was observed between the frequency of clonal seeds and seed-setting, with clonal lines with high frequency of seed-setting rate exhibiting low frequency of clonal seeds, and vice versa (Fig. 1h, Table 2B). This phenomenon is prevalent in all clonal lines we have obtained (Table 2). Fortunately, we can obtain the relatively high frequency of clonal seeds in the case of ensuring the seed-setting rates.

All progenies of the p24MiMe T_0_ events were determined to be tetraploid (Table 1), a result of diploid gamete fusion. If parthenogenesis is triggered through the expression of *BBM1*, driven by *AtDD45* in combination with *MiMe*, clonal seeds of the T_1_ progeny should be shown as previously at a frequency of 10–30% (Khanday et al. 2019). Exceeding our expectations, sg*MiMe*_*pAtDD45:BBM1* (p63C) transformants produced over 90.00% diploid clonal plants, exhibiting seed-setting rates were 44.88% and 51.35% (Table 1, Table 2A). As for the transformants of p94C, the maximum frequency of clonal seeds reached 97.50% (line HW7), while the peak seed-setting rate was 68.16% (line HW14). Notably, the line HW14 showed the most optimal comprehensive traits, with a clonal seeds rate of 90.79% and a seed-setting rate of 68.16% (Table 2A). In the T_0_ transformants of p95C, 11 of the 25 were analyzed, the maximum frequency of clonal seeds reached 98.21% (line CL10), and the peak seed-setting rate was 83.67% (line CL3), which was comparable to that of wild-type (88.65%, range 84.17–93.10%). Notably, the line CL3, exhibiting superior comprehensive traits, demonstrated 46.48% of clonal seeds and 83.67% of seed-setting rate (Table 1, Table 2B). The results suggest that an unexpectedly high frequency of clonal seeds can also be yielded using *AtDD45* promoter to drive *BBM1* expression or fusion promoter in combination with the *MiMe*.

**Table 1:** Frequency of diploid clonal plants in T_1_ plants of p24MiMe, p63C, p94C, and p95C

T-DNA constructs	Events	Progeny tested	Diploids	Tetraploids	% Clonal seeds
sg*MiMe*	MM-9	20^a^	0	20	0
(p24MiMe)	MM-10	18^a^	0	18	0
	MM-11	23^a^	0	23	0
sg*MiMe*_*pAtDD45*:*BBM1*	G23-6	20^b^	18	2	90.00
(p63C)	G23-18	58^a^	56	2	96.55
‘sg*MiMe*’_*pAtDD45*:*BBM1*	HW7	80^a^	78	2	97.50
(p94C)	HW10	80^a^	53	27	66.25
	HW11	84^a^	69	15	82.14
	HW14	76^a^	69	7	90.79
	HW16	107^a^	99	8	92.52
‘sg*MiMe*’_*pAtMYB98*^*+*^* pAtDD1*	CL1	88^a^	8	80	9.09
^*+*^*pOsECA1-like1*:*WUS*	CL3	71^a^	33	38	46.48
_*pAtDD45*:*BBM1* (p95C)	CL6	40^a^	32	8	80.00
	CL8	56^a^	41	15	73.21
	CL9	56^a^	3	53	5.36
	CL10	56^a^	55	1	98.21
	CL11	16^a^	14	2	87.50
	CL12	32^a^	30	2	93.75
	CL13	32^a^	30	2	93.75
	CL14	80^a^	51	29	63.75
	CL15	72^a^	26	46	36.11

^a^Ploidy level was determined through phenotypic observation and subsequently confirmed by flow cytometry (*n* = 8)

^b^Ploidy level was determined by flow cytometry analysis

**Table 2:** Clonal seeds rate and seed-setting rate in T_1_ and T_2_ plants of p63C, p94C, and T_1_ plants p95C

A					
T-DNA constructs	Events	T_1_		T_2_	
		% Clonal seeds	% Seed-setting	% Clonal seeds	% Seed-setting
sg*MiMe*_*pAtDD45*:*BBM1*	WT	0.00	76.26 ± 11.22a	0.00	76.26 ± 11.22a
(p63C)	G23-6	90.00	44.88 ± 5.30b	90.63	55.05 ± 4.58b
	G23-18	96.55	51.35 ± 4.27b	15.18	56.92 ± 8.59b
‘sg*MiMe*’_*pAtDD45*:*BBM1*	WT	0.00	88.65 ± 4.47a	0.00	88.65 ± 4.47a
(p94C)	HW7	97.50	33.73 ± 0.36e	98.70	30.89 ± 0.58d
	HW10	66.25	41.03 ± 2.44d	38.75	56.21 ± 0.75c
	HW11	82.14	54.15 ± 4.89c	77.57	55.84 ± 1.54c
	HW14	90.79	68.16 ± 2.17b	89.39	66.10 ± 10.01b
	HW16	92.52	41.86 ± 2.81d	94.38	49.39 ± 3.69c

B			
T-DNA constructs	Events	% Clonal seeds	% Seed-setting
‘sg*MiMe*’_*pAtMYB98*^*+*^* pAtDD1*	WT	0.00	88.65 ± 4.47a
^*+*^*pOsECA1-like1*:*WUS*	CL1	9.09	75.71 ± 2.99b
_*pAtDD45*:*BBM1* (p95C)	CL3	46.48	83.67 ± 2.65a
	CL6	80.00	42.99 ± 2.56d
	CL8	73.21	39.72 ± 3.75d
	CL9	5.36	61.59 ± 0.41c
	CL10	98.21	15.75 ± 4.30f
	CL11	87.50	27.96 ± 2.45e
	CL12	93.75	20.69 ± 9.77f
	CL13	93.75	29.06 ± 2.51e
	CL14	63.75	59.70 ± 2.49c
	CL15	36.11	60.28 ± 4.97c

Data were means (*n* = 3, Duncan’s test with a significance of 0.05%)

To determine if heterozygosity was maintained in the progenies of apomicts, pinpoint sequencing genotyping was conducted in the wild-type, F_2_ progenies, and five T_1_ clonal lines (Fig. 1k). Compared to wild-type, the site similarity in F_2_ progenies ranged from 43.62% to 56.44%, arising from meiotic crossovers. In contrast, clonal lines exhibited a site similarity of up to 98.92%, due to the absence of meiotic division. The sequencing result indicates that the T_1_ clonal lines have retained their heterozygous genotypes, confirming their clonal nature.

The phenotypes are important to investigate whether clonal lines are suitable for commercial applications. We selected four clonal lines of the p94C, all of which yielded more than 80.00% diploid clonal plants (T_1_), to explore if these clonal plants maintain the F_1_ hybrid (wild-type) phenotype. The gross phenotype of T_1_ clonal plants displayed similar traits such as plant height, number of panicles, and panicle length with the F_1_ hybrid plants (Fig. 1i–j). Phenotypes were more thoroughly examined in T_1_ progenies in four p94C transformants, grown with control wild-type. No significant differences were detected in the number of panicles and grain width when comparing the control F_1_ hybrid with the clonal plants. The few exceptions comprised a significantly higher plant height and longer grain length in line HW14 and lighter one-thousand grain weight in line HW7 and shorter panicle length in all clonal lines except line HW14 (Table S2). These differences may arise from somatic mutations and environmental situations. In summary, the assessment of observable characteristics in progenies from p94C transformants reveals a consistent phenotype closely resembling that of the F_1_ hybrid.

Altogether, using *AtDD45* and fusion promoter can yield one-line hybrid rice with high-frequency clonal seeds and near-normal fertility in combination with the *MiMe*. The genotype and phenotype are retained in the offspring of clonal plants.

### High frequency of multiple-embryos resulted from the ectopic expression of *BBM1* combined with *MiMe*

**Fig. 2:**
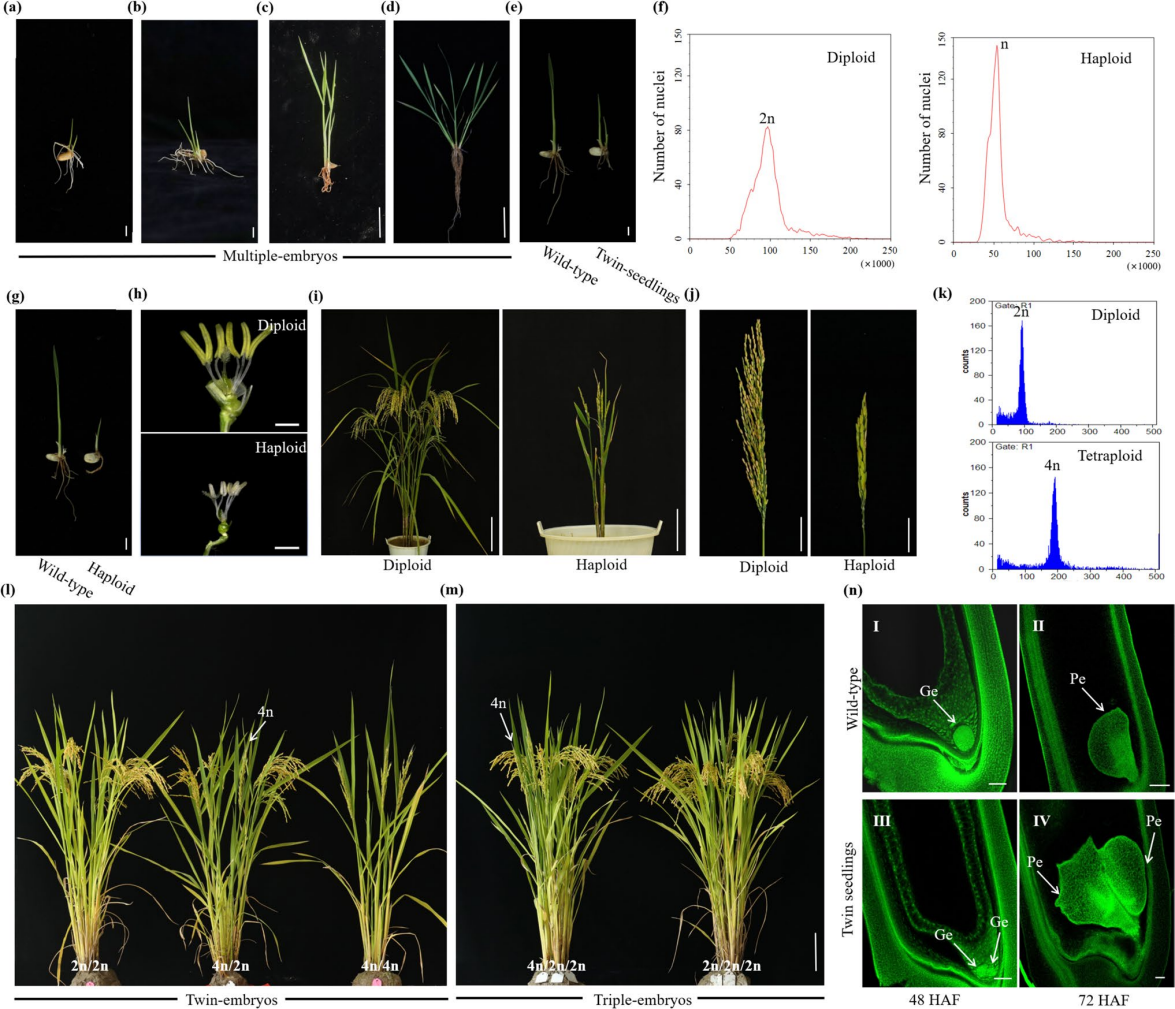
Ploidy, phenotype, and embryo sac observation of multiple-embryos. **a**, **b** Discovery of multiple-embryos. In terms of morphology, the multiple-embryos displayed a single seed with two or three plumule axes. Scale bars, 2 mm. **c**, **d** Morphology of multiple-embryos. At 15 d after germination, the morphology of multiple-embryos showed two or three seedlings. Scale bars, 2 cm. **e** Discovery of multiple-embryos in p63C transgenic plants (without *MiMe* mutation). Germination of 6 d-old twin-embryos. Scale bars, 2 mm. **f** Flow cytometry DNA histograms for ploidy determination of multiple-embryos (2n/n). One was a sexual diploid showing a 2n peak, and the other was an asexual haploid showing a 1n peak. **g** Characterization of haploids. Differences in height between wild-type and haploid seedlings derived from p63C transformants (without *MiMe* mutation) at 6 d after germination. Scale bars, 2 mm. **h** Comparative analysis of the morphology of diploid and haploid floral organs. Scale bars, 1 mm. **i** Comparison of the morphology across different ploidy (2n/n) in multiple-embryos. Two seedlings were planted separately because the asexual haploid had limited growth potential. Scale bars, 20 cm. **j** Comparison of the main spikes in multiple-embryos (2n/n). Compared to the sexual diploid, the panicle of the asexual haploid showed infertile seeds. Scale bars, 5 cm. **k** Ploidy analysis of the multiple-embryos (2n/4n) according to flow cytometry. One of the multiple-embryos was diploid (asexual), showing a 2n peak; and the other was tetraploid (sexual), showing a 4n peak. **l** Comparison of the morphology across different ploidy (2n/2n, 4n/2n, and 4n/4n) in twin-embryos. Scale bars, 20 cm. **m** Comparison of the morphology across different ploidy (4n/2n/2n and 2n/2n/2n) in triple-embryos. Scale bars, 20 cm. **n** Representative embryos at 48 h and 72 h after flowering (HAF) from wild-type (I, II) and multiple-embryos (III, IV). Compared to wild-type, multiple-seedlings showed two embryos formed at the micropyle. *Ge* globular embryos, *Pe* pyriform embryos. Scale bars, 2 mm

In the clonal lines, there was a phenomenon where a single seed with multiple plumule axis, typically two or three, which we have termed multiple-embryos (Fig. 2a–d). Among the T_0_ transformants, two of the p63C, four of the p94C, and six of the p95C exhibited over 20.00% of multiple-embryos, and this phenomenon was maintained in descendants (Table 3, Table S3). Significantly, line HW7 (p94C) displayed a high frequency (60.99%) of multiple-embryos (Table 3). In addition, all these transformants also showed a high penetrance of apomixis (Table 1). Unsurprisingly, no multiple-embryos were observed in *MiMe*-only mutants (line MM-9, MM-10 and MM-11 from p24MiMe). To determine if the ectopic expression of the *BBM1* gene in combination with *MiMe* induced multiple-embryos, we examined four transformants without the *MiMe* mutation (lines G23-5, G23-11, G23-13, and G23-16 from p63C). Fewer than 4.00% of multiple-embryos were exhibited in the transformants (Fig. 2e, Table 3), which was consistent with our expectations. These findings show that ectopic expression of *BBM1* can induce fewer multiple-embryos, but the high penetrance of multiple-embryos resulted from the combination of ectopic expression of *BBM1* with *MiMe*.

**Table 3:** Frequency of multiple-embryos in T_1_ plants of p24MiMe, p63C, p94C, and p95C

T-DNA constructs	Events	Progeny tested	Multiple-embryos	Single-embryos	% Multiple-embryos
sg*MiMe*	MM-9	50	0	50	0
(p24MiMe)	MM-10	62	0	62	0
	MM-11	33	0	33	0
sg*MiMe*_*pAtDD45*:*BBM1*	G23-6	135	29	106	21.48
(p63C)	G23-18	146	46	100	31.51
	G23-5^a^	207	3	204	1.45
	G23-11^a^	47	1	46	2.13
	G23-13^a^	104	2	102	1.92
	G23-16^a^	78	3	75	3.85
‘sg*MiMe*’_*pAtDD45*:*BBM1*	HW7	282	172	110	60.99
(p94C)	HW10	90	9	81	10.00
	HW11	171	38	133	22.22
	HW14	271	104	167	38.38
	HW16	298	60	238	20.13
‘sg*MiMe*’_*pAtMYB98*^*+*^* pAtDD1*	CL1	89	3	86	3.37
^*+*^*pOsECA1-like1*: *WUS*	CL3	74	6	68	8.11
_*pAtDD45*:*BBM1* (p95C)	CL6	47	12	35	25.53
	CL8	65	13	52	20.00
	CL9	58	0	58	0.00
	CL10	64	19	45	29.69
	CL11	25	6	19	24.00
	CL12	47	21	26	44.68
	CL13	46	10	36	21.74
	CL14	82	0	82	0.00
	CL15	74	0	74	0.00

^a^Events G23-5, 11, 13 and 16 were transformants of p63C (without *MiMe* mutation)

The ploidy level of multiple-embryos was determined through phenotypic observation and confirmed using flow cytometry. Ploidy analysis showed that twin-embryos from transformants without *MiMe* mutation were 2n/n chimeric seedlings (Fig. 2f). Compared to sexual diploid plants, asexual haploids showed typical haploid characteristics, including sterile and small floral organs (Fig. 2g–j). In contrast, twin-embryos from transformants (*BBM1* expression with *MiMe* mutation) were either both 2n, both 4n, or 4n/2n chimeric seedlings (Fig. 2k–l). The triple-embryos exhibited 4n/2n/2n or 2n/2n/2n (Fig. 2m). These results suggest that 2n seedlings may arise from apogamy while 4n seedlings result from zygotes, indicating a potential to increase the frequency of clonal seeds via apogamy embryogenesis.

We employed whole-mount stain-clearing laser scanning confocal microscopy (WCLSM) to observe the development of embryo sacs in diploid clonal plants. Notably, at 48 and 72 h after flowering (HAF), we observed two embryos forming at the micropyle in embryo sacs. In contrast, the control wild-type displayed only a single embryo (Fig. 2n). This suggests that the presence of multiple seedlings might arise from two or more embryos in the same embryo sac, sharing a common endosperm.

### High efficiency of apomixis was atavism (transmission through skipped generations)

The stability of the synthetic apomixis system over multiple generations was discovered in the majority of transgenic lines. In the T_2_ plants of line G23-6 (p63C), the determination of ploidy level revealed more than 90.00% of clonal plants, consistent with observations in T_1_ plants (Table 1, Table S3). Similarly, among the T_2_ clonal lines of p94C, 4 out of the 5 apomicts exhibited clonal seeds frequencies ranged from 77.57% to 98.70%, which were also consistent with the performance of their T_1_ plants (Table 1, Table S3). The seed-setting rates of T_2_ diploid clonal plants from p63C (range 55.05–56.92%) and p94C (range 30.89–66.10%) transformants were corresponding to observations in T_1_ plants (Table 2A). Altogether, both apomixis and seed-setting rates exhibited stability across generations in the majority of transgenic lines.

**Fig. 3:**
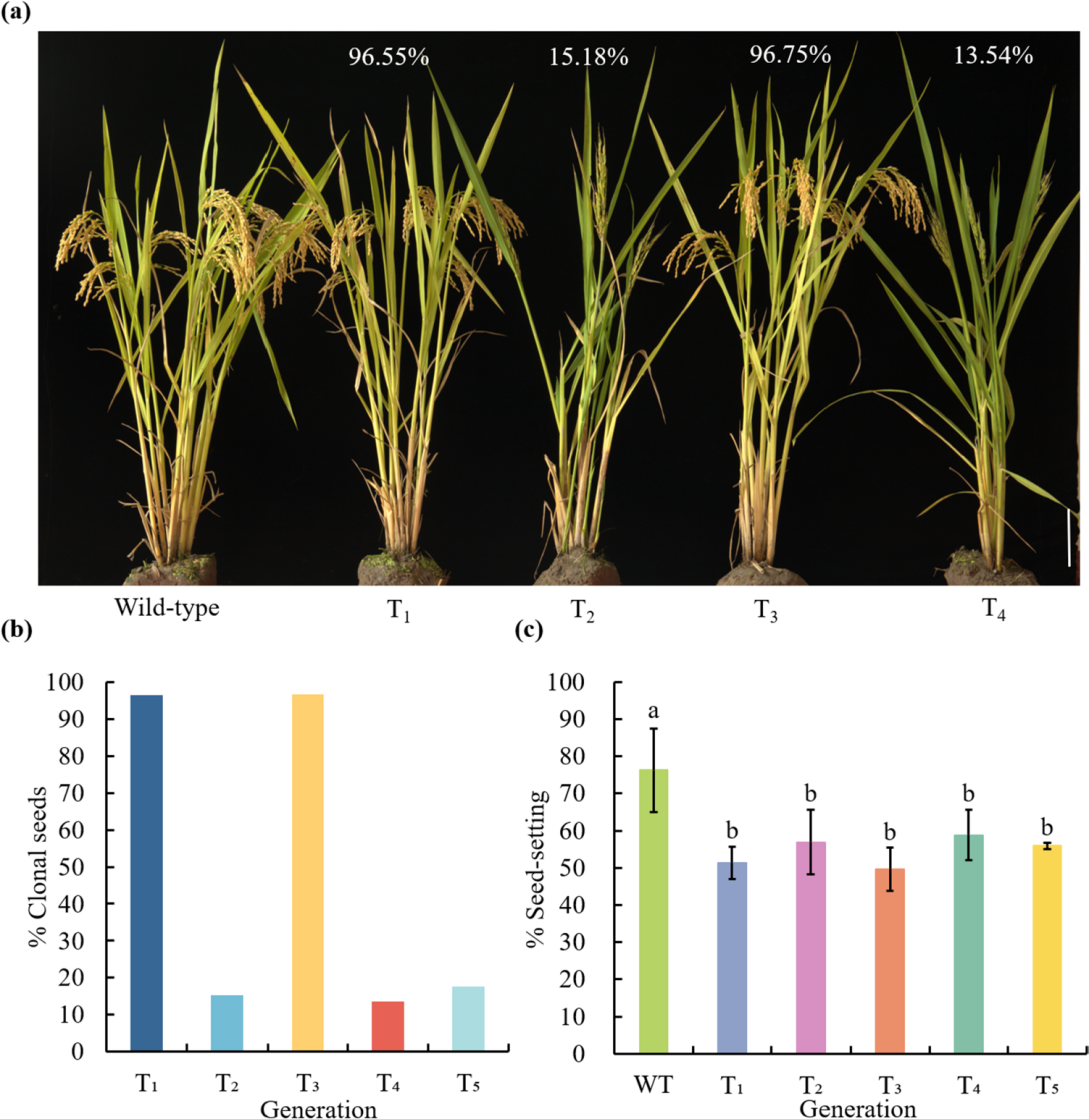
The phenomenon of atavism. **a** Frequency of clonal seeds in T_1_, T_2_, T_3_ and T_4_ plants of line G23-18 (p63C). **b** Comparison of frequency of clonal seeds from T_1_ to T_5_ plants in line G23-18 (p63C). In the T_1_, T_2_, T_3_, T_4,_ and T_5_ generations, the average frequency of diploid clonal plants was 96.55%, 15.18%, 96.75%, 13.54%, and 17.56%, respectively. Scale bars, 20 cm. Details were provided in Table S3. **c** Seed-setting rate in T_1_ to T_5_ clonal plants of line G23-18 (p63C). In the T_1_, T_2_, T_3_, T_4_ and T_5_ generations, the average seed-setting rate was 51.35%, 56.92%, 49.64%, 58.82%, and 55.88%, respectively

Interestingly, the phenomenon of atavism (transmission across skipped generations) was observed in two lines based on the frequency of apomixis. In line G23-18 (p63C), the clonal seeds frequencies for T_1_ through T_5_ plants were 96.55%, 15.18%, 96.75%, 13.54%, and 17.56%, respectively (Table 1, Table S3). Notably, the frequency was increased in T_1_ and T_3_ plants but decreased in T_2_ and T_4_ plants. This atavism phenomenon was absent in the T_5_ generation (Fig. 3a, b). The seed-setting rate of T_1_ to T_5_ clonal plants from line G23-18 (p63C) was significantly lower than that of the wild-type (76.26%), with the average seed-setting rates of 51.35%, 56.92%, 49.64%, 58.82%, and 55.88%, respectively (Fig. 3c). The similar result was also observed in line HW10 (p94C), with the apomixis frequencies of 66.25% and 38.75% (Table 1, Table S3), and the seed-setting rates of 66.25% and 56.21% in T_1_ and T_2_ plants (Table 2), respectively. These findings show a trend of high frequency of clonal seeds in alternate generations, referred to as atavism.

## Discussion

One-line hybrid rice is Professor Longping Yuan’s living will and unfinished business. In 1987, he proposed a development strategy for hybrid rice: from a three-line to a two-line, and later on to a one-line, paralleling a shift from complexity to simplicity (Yuan 1987). The one-line hybrid rice could be achieved by synthetic apomixis, which can lead to the fixation of heterosis and reduce the cost of hybrid rice seed production. A previous study using the promoter *AtDD45* to drive *BBM1* just reached 29.20% synthetic apomixis in the inbred cultivar Kitaake (Khanday et al. 2019). In this study, we used *AtDD45* promoter to drive *BBM1* expression in combination with the *MiMe*, resulting in the production of high-efficiency clonal seeds at a rate of 98.70% in hybrid rice YY4 (japonica/indica). In addition, we designed the construct ‘sg*MiMe*’_*pAtMYB98_pAtDD1_pOsECA1*-*like1:WUS*_*pAtDD45:BBM1*, yielding clonal seeds frequencies of up to 98.21%. We also analyzed the differences in fertility between the control and clonal lines under a field trial. The clonal lines of p94C (range 33.73–68.16%) and p95C (range 15.75–83.67%) exhibited highly variable seed-setting rates under a field trial. The highest seed-setting rate was comparable to the control YY4 hybrid (88.65%). Specifically, the lines with the most optimal comprehensive traits were line HW14 (p94C) and line CL3 (p95C), the seed-setting rate of line HW14 and line CL3 was 68.16% and 83.67%, respectively. Meanwhile, the production of clonal seeds reached 90.79% for line HW14 and 46.48% for line CL3. These results indicate the potential of synthetic apomixis in agricultural applications.

In addition, we observed a conflicting relationship between the frequency of seed-setting and clonal seeds in diploid clonal plants, where clonal lines with a high frequency of seed-setting rate exhibited a low frequency of clonal seeds, and vice versa. We speculate that during the initiation of embryo autonomous development through parthenogenesis of synthetic apomixis, signals for cell division and differentiation are released at high penetrance. Consequently, other cells in the embryo sac, such as the synergid cells, perceive these signals as an indication of completed fertilization, which will affect sperm cell entry into the embryo sac, subsequently influencing central cell fertilization and inhibiting endosperm formation, thereby leading to a reduced seed-setting rate. An autonomous endosperm may result in both normal seed formation and an increase in the seed-setting rate. Another strategy to increase the seed-setting rate is employing egg-cell-specific promoters, which activate expression at the exact time to induce embryogenesis without affecting central cell fertilization. We also propose an alternative way, sorting the clonal seeds, to address the conflict between the frequency of seed-setting and clonal seeds. In our research, we obtained some lines (e.g., line CL3) with near-normal seed-setting rates, and their apomixis efficiency was relatively low. Subsequently, we can separate the clonal seeds using pollen-specific gene switch system. This technology distinguishes non-fluorescent clonal seeds from red-fluorescent sexual seeds through photoelectric sorting, such that the clonal seeds could be selectively used for field planting (Cao et al. 2019). We have reported that clonal lines exhibited a high penetrance of multiple-embryos for the first time. Specifically, two embryos were observed in the embryo sac and this trait was stably inherited across multiple generations. No multiple-embryos were observed in *MiMe*-only mutants, and they were low frequency in the transformants without *MiMe* mutation from p63C. This indicate that the *BBM1* gene is essential for the production of multiple embryos. *MiMe* mutants produced diploid gametes, and the ploidy detection of multiple-embryos was 2n/2n, 4n/4n, or 4n/2n chimeric plants, respectively. Ploidy detection of multiple-embryos in the transformants without *MiMe* mutation was 2n/n chimeric plants, due to haploid gametes. In the embryo sac, asexual haploid embryos exhibit reduced viability in competing with sexual diploid embryos. This could account for the observation that only the combination of *MiMe* and the ectopic expression of *BBM1* results in the production of multiple-embryos at a high efficiency. We also detected haploids in the transformants without *MiMe* mutation with frequencies less than 8.33% (Table S4). We speculate that the formation of multiple embryos in clonal lines can be attributed to the following reasons: the first is embryo division, *BBM1* protein activates the gene involved in embryo division; the second is that promoter *AtDD45* is not only expressed in egg cells but also other cells, such as synergids, which may subsequently develop into embryos; the third is *BBM1* protein activates the gene involved in cell division during egg cell formation resulted in two egg cells in the micropyle. As a result, one seed with two or three embryos, and germinating multiple-embryos, it is of great significance to the innovation and utilization of crop germplasm, with fewer seeds to obtain the same yielding.

The stability of clonal lines over generations has been previously demonstrated in previous reports (Vernet et al. 2022; Liu et al. 2023). Our study was consistent with previous research in the majority of lines. An intriguing phenomenon of atavism was observed in two lines, with high penetrance of apomixis in both the T_1_ and T_3_ generations, contrasting with low occurrences in the T_2_ and T_4_ generations. Notably, the atavism phenomenon disappeared in the T_5_ generation. This observation has not been previously reported and contradicts traditional genetic principles. Epigenetic control of gene expression may be responsible for this phenomenon, and further investigation into the underlying mechanisms is warranted.

### Supplementary Information

Below is the link to the electronic supplementary material.

Supplementary file1 (DOCX 44 KB)

## Data Availability

The datasets generated during and/or analyzed during the current study are available from the corresponding author on reasonable request.
