# Supplementary material for: One-line hybrid rice with high-efficiency synthetic apomixis and near-normal fertility
Source: Plant Cell Rep. 2024 Feb 24;43(3):79. doi: 10.1007/s00299-024-03154-6 (PMC10894110; doi:10.1007/s00299-024-03154-6)
Supplement: Supplementary file 1 — Supplementary file1 (DOCX 44 KB) [file 299_2024_3154_MOESM1_ESM.docx]

**Supplementary Materials**

**Table S1. Mutations around the targets of *OsOSD1*, *PAIR1* and *OsREC8* in p24MiMe, p63C, p94C, and p95C transformants****.**

**A.**

| T-DNA  construct | Events | *OsOSD1* gRNA-1 | *OsOSD1* gRNA-2 | *PAIR1* gRNA-1 | *PAIR1* gRNA-2 | *OsREC8* gRNA-1 | *OsREC8* gRNA-2 |
| --- | --- | --- | --- | --- | --- | --- | --- |
| sg*MiMe* | MM-9 | Homozygous | WT | WT | Homozygous | WT | Homozygous |
| (p24MiMe) | MM-10 | Homozygous | WT | WT | Biallelic | WT | Homozygous |
|  | MM-11 | Homozygous | WT | WT | Biallelic | WT | Homozygous |
| sg*MiMe*_ *pAtDD45*: | G23-6 | Biallelic | WT | WT | Biallelic | WT | Biallelic |
| *BBM1* (p63C) | G23-18 | Biallelic | WT | WT | Biallelic | WT | Biallelic |

**B.**

| T-DNA  constructs | Events | *OsOSD1*  gRNA-1 | *PAIR1*  gRNA-3 | *OsREC8*  gRNA-1 |
| --- | --- | --- | --- | --- |
| ‘sg*MiMe*’_*pAtDD45:* | HW7 | Biallelic | Biallelic | Biallelic |
| *BBM1* | HW10 | Biallelic | Biallelic | Biallelic |
| (p94C) | HW11 | Biallelic | Biallelic | Biallelic |
|  | HW14 | Biallelic | Biallelic | Biallelic |
|  | HW16 | Homozygous | Biallelic | Homozygous |
| ‘sg*MiMe*’_*pAtMYB98*+*pAtDD1* | CL1 | Biallelic | Homozygous | Biallelic |
| +*pOsECA1-like1*:*WUS* | CL3 | Biallelic | Homozygous | Biallelic |
| _*pAtDD45*:*BBM1* (p95C) | CL10 | Biallelic | Homozygous | Biallelic |
|  | CL12 | Biallelic | Biallelic | Biallelic |
|  | CL13 | Biallelic | Homozygous | Biallelic |

Biallelic, two alleles were mutated, but the mutation type was different.

Homozygous, two alleles were mutated with the same mutation type.

**Table S2.** **Agronomic characters of T_1_ diploid clonal plants of p94C.**

| T_1_ plants | Plant height (cm) | Number of panicles | Panicle length (cm) | 1000 grain weight  (g) | Grain length  (mm) | Grain width  (mm) |
| --- | --- | --- | --- | --- | --- | --- |
| WT | 76.54±  2.40b | 5.50±  2.17a | 19.21±  1.04a | 24.50±  0.85a | 7.06±  0.06b | 3.08±  0.04a |
| HW7 | 76.29±  2.18b | 6.33±  1.03a | 17.91±  0.81b | 23.37±  1.35b | 7.01±  0.11b | 3.03±  0.03a |
| HW11 | 73.00±  2.24b | 6.17±  1.17a | 17.43±  0.50b | 24.63±  0.33a | 7.04±  0.08b | 3.03±  0.03a |
| HW14 | 82.99±  4.41a | 7.25±  2.80a | 18.82±  0.77a | 24.99±  0.79a | 7.22±  0.06a | 3.05±  0.04a |
| HW16 | 74.60±  2.81b | 5.67±  1.86a | 17.29±  0.81b | 24.33±  0.48a | 7.07±  0.06b | 3.05±  0.01a |

**Table S3. Detail of diploid clonal plants and multiple-embryos** **of p63C and p94C.**

| T-DNA |  | Lines | | Apomixis | |  |  | Multiple-embryos | |  |  |
| --- | --- | --- | --- | --- | --- | --- | --- | --- | --- | --- | --- |
| constructs |  |  | | Plants tested | Diploids | Tetraploids | % Clonal seeds | Plants tested | Multiple-embryos | Single- embryos | % Multiple-embryos |
| sg*MiMe*_ | T_2_ | | G23-6/8 | 92^#^ | 85 | 7 | 92.39 | 107 | 50 | 57 | 46.73 |
| *pAtDD45*: |  | | G23-6/9 | 100^#^ | 89 | 11 | 89.00 | 130 | 59 | 71 | 45.38 |
| *BBM1* |  | | Average | 192 | 174 | 18 | 90.63 | 237 | 109 | 128 | 45.99 |
| (p63C) |  | | G23-18/1 | 33^#^ | 4 | 29 | 12.12 | 40 | 17 | 23 | 42.50 |
|  |  | | G23-18/4 | 110^#^ | 10 | 100 | 9.09 | 139 | 14 | 125 | 10.07 |
|  |  | | G23-18/6 | 160^#^ | 32 | 128 | 20.00 | 169 | 20 | 149 | 11.83 |
|  |  | | Average | 303 | 46 | 257 | 15.18 | 348 | 51 | 297 | 14.66 |
| ‘sg*MiMe*’_ | T_2_ | | HW7/11 | 231^#^ | 228 | 3 | 98.70 | 257 | 165 | 92 | 64.20 |
| *pAtDD45:* |  | | HW10/7 | 80^#^ | 31 | 49 | 38.75 | 149 | 19 | 130 | 12.75 |
| *BBM1* |  | | HW11/9 | 107^#^ | 83 | 24 | 77.57 | 132 | 20 | 112 | 15.15 |
| (p94C) |  | | HW14/28 | 66^#^ | 59 | 7 | 89.39 | 119 | 47 | 72 | 39.50 |
|  |  | | HW16/17 | 178^#^ | 168 | 10 | 94.38 | 180 | 46 | 134 | 25.56 |
|  |  | | Average | 662 | 569 | 93 | 85.95 | 837 | 297 | 540 | 35.48 |
| sg*MiMe*_ | T_3_ | | G23-18/1/1 | 23* | 23 | 0 | 100.00 | 30 | 14 | 16 | 46.67 |
| *pAtDD45*: |  | | G23-18/1/3 | 195^#^ | 195 | 0 | 100.00 | 205 | 143 | 62 | 69.76 |
| *BBM1* |  | | G23-18/1/6 | 16* | 13 | 3 | 81.25 | 33 | 16 | 17 | 48.48 |
| (p63C) |  | | G23-18/4/1 | 106^#^ | 102 | 4 | 96.23 | 120 | 81 | 39 | 67.50 |
|  |  | | G23-18/4/4 | 46^#^ | 43 | 3 | 93.48 | 50 | 31 | 19 | 62.00 |
|  |  | | G23-18/4/7 | 137^#^ | 130 | 7 | 94.89 | 140 | 79 | 61 | 56.43 |
|  |  | | Average | 523 | 506 | 17 | 96.75 | 578 | 364 | 214 | 62.98 |
|  | T_4_ | | G23-18/1/1/2 | 61^#^ | 13 | 48 | 21.31 | 335 | 12 | 323 | 3.58 |
|  |  | | G23-18/4/1/9 | 131^#^ | 13 | 118 | 9.92 | 179 | 15 | 164 | 8.38 |
|  |  | | Average | 192 | 26 | 166 | 13.54 | 514 | 27 | 487 | 5.25 |
|  | T_5_ | | G23-18/1/1/2/1 | 102^#^ | 23 | 79 | 22.55 | 118 | 5 | 113 | 4.24 |
|  |  | | G23-18/4/1/9/4 | 103^#^ | 13 | 90 | 12.62 | 122 | 2 | 120 | 1.64 |
|  |  | | Average | 205 | 36 | 169 | 17.56 | 240 | 7 | 233 | 2.92 |

^*^ Ploidy level was determined by flow cytometry analysis.

^#^ Ploidy level was determined through phenotypic observation and subsequently confirmed by flow cytometry (n=8).

**Table S4. Haploid induction in p63C transformants (without *MiMe* mutation).**

| Events | Progeny tested | Haploids | Diploids | % Haploids |
| --- | --- | --- | --- | --- |
| G23-11^a^ | 14 | 1 | 13 | 7.14 |
| G23-13^a^ | 12 | 1 | 11 | 8.33 |
| Average | 26 | 2 | 24 | 7.69 |

^a^ Line G23-11 and G23-13, both transformants without *MiMe* mutation, were selected for analysis as just harboring the *pAtDD45:BBM1* T-DNA.

**Table S5. Target sequences in *OsOSD1*, *PAIR1* and *OsREC8* gene.**

| sgRNA | 5'-3' target sequence |
| --- | --- |
| *OsOSD1* gRNA-1 | AGGGCGGCGGCGCTCGCCGACCCCTCGGGTGG |
| *OsOSD1* gRNA-2 | CGGGCCCTGCCGCCGACGAGCAACAAGG |
| *PAIR1* gRNA-1 | AGGTCTCCCTCGACGACAACCTCCTCACC |
| *PAIR1* gRNA-2 | ATTACCAAGCAACCCAGTGCACCGCTGG |
| *PAIR1* gRNA-3 | CGTCGGCTTCCGGTTCCGTGGCGGTGGCGGTG |
| *OsREC8* gRNA-1 | TAGGTGGTGTGGCGATCGTGTACGAGAGG |
| *OsREC8* gRNA-2 | TCGGTACCCATGGCACTAAGGCTCTCCG |

The target sequence of wild-type and PAM is in blue and red, respectively.

**Table S6. The list of primers for detection of *OsOSD1*, *PAIR1*, and *OsREC8* target sites.**

| Primer | Primer sequence (5'-3') | Product size |
| --- | --- | --- |
| *OsOSD1*-F1 | TTACTTGGAAGAGGCAGGAGCC | 364bp |
| *OsOSD1*-R1 | ACCTTGACGACTGACGTGATGTC |  |
| *OsOSD1*-F2 | ATCTCCAGGATGCCTGAAGTGAG | 584bp |
| *OsOSD1*-R2 | CCTAGACTGCTACTCTTGCTAGTGAT |  |
| *PAIR1*-F1 | GTGGTGTGGTGTGTTCAGGAG | 344bp |
| *PAIR1*-R1 | TGGAATCCCCAATCAGTAAGGCAC |  |
| *PAIR1*-F2 | CTGTACCTGTGCATCTAATTACAG | 594bp |
| *PAIR1*-R2 | CCCCATCTTATGTACTGAGCTTGCCAG |  |
| *PAIR1*-F3 | CGAAGGAGAAGGCTACGGC | 511bp |
| *PAIR1*-R3 | CAGGGACAGGAGTGAGTGGAA |  |
| *OsREC8*-F1 | GCACTAAGGCTCTCCGGAATTCTC | 326bp |
| *OsREC8*-R1 | AATGGATCAAGGAGGAGGCACC |  |
| *OsREC8*-F2 | GCGACGCTTCACTCGAAGATCA | 596bp |
| *OsREC8*-R2 | CGCCATGCCTCGTTGATCTCAA |  |
